# Relationships Between Intrinsic Religiosity, Stress, Anxiety and Depression in Outpatients and Health Care Professionals in Brazil

**DOI:** 10.1007/s10943-026-02611-8

**Published:** 2026-03-14

**Authors:** Élida Mara Carneiro

**Affiliations:** 1 Integrative and Complementary Practices Center – NUPIC, Clinics Hospital, 141, José Pimenta Camargo Street, Uberaba, MG Brazil; 2 Clinics Hospital, Federal University of Triângulo Mineiro (UFTM), 330, Getúlio Guaritá Street, Uberaba, MG 38025-440 Brazil

**Keywords:** Religion, Spirituality, Stress, Anxiety, Depression

## Abstract

Religiosity and Spirituality (R/S) have been highly studied in connection with mental health, but has hardly been explored in outpatients and health care professionals (HCP). This study aimed to evaluate intrinsic religiosity (IR) and its relationship with mental health in outpatients and HCP in Brazil. A total of 1.864 participants were evaluated. Based on a linear regression model, stress was associated with lower IR, age and education (*p* < 0.05); anxiety was associated with lower IR, age and unemployment (*p* < 0.05); and depression was associated with lower IR, age and education (*p* < 0.05) in outpatients. In HCP, stress was associated with lower IR (*p* < 0.05); women and profession were associated with anxiety (*p* < 0.05); and lower IR and women were associated with depression (*p* < 0.05). Regardless of the group they were in, higher IR was found to be associated with less stress and depression symptoms, as well as less anxiety symptoms in outpatients. These findings support the relevance of R/S for improving mental health in outpatients and HCP. The HCP and managers should be aware of the beliefs of their patients, and encourage their participation in R/S strategies.

## Introduction

Research on the effects of religiosity and spirituality (R/S) on mental health has grown rapidly (Lucchetti et al., 2021; Rezende-Pinto & Moreira-Almeida, 2023). Religiosity has been described as a protective factor against depression (Miller et al., 2012).

Studies have shown that religiosity can have a positive effect on the clinical evolution of patients and improve health behaviors (Rezende-Pinto & Moreira-Almeida, 2023). Furthermore, religiosity can alleviate anxiety and depressive symptoms, among other benefits (de Diego-Cordero et al., 2022; Fenelon & Danielsen, 2016; VanderWeele et al., 2017).

Intrinsic religiosity (IR) specifically has been associated with resilience, fewer suicide attempts in severely depressed inpatients, better immunological parameters, and lower mortality over 4 years in patients living with HIV (Carneiro et al., 2022; Mosqueiro et al., 2021).

A meta-analysis including longitudinal studies revealed evidence for a positive effect of R/S on mental health, but this effect was small (Smith et al., 2003). Therefore, it is necessary to identify the impact of R/S on stress, anxiety, and depression in outpatients and health care professionals (HCP). The present study aimed to evaluate IR and its relationship with mental health in outpatients and HCP in Brazil.

## Methods

### Study Design

The present study is part of a larger research project that seeks to investigate the impact of R/S in the context of health in individuals of the population at Clinics Hospital (CH); Maria da Glória (MG), Specialty, Chemotherapy, and Integrative Practices Center Outpatient Clinics of the Federal University of Triângulo Mineiro (UFTM), Brazil. These places provide services to 27 cities registered by Regional Health Superintendence of Uberaba, MG.

### Ethical Issues

The present study complied with resolution no. 1.692.927 from the Brazilian National Health Council and was duly approved by the Research Ethics Committee at UFTM. All participants read and signed an informed consent form agreeing to voluntary participation.

### Eligibility Criteria and Study Participants

Individuals who met all the inclusion and exclusion criteria were included in the study. For outpatients, the inclusion criteria included being treated at Outpatient Clinics with the ability to understand and answer the questionnaires. The exclusion criterion was age under 18 years, both sexes.

For HCP, the inclusion criteria included being a professional at CH and Outpatient Clinics; age above 18 years, both sexes.

### Study Setting and Data Collection

The recruitment strategy used was online data collection through the Google forms and face-to-face data collection. The professionals were recruited by invitation to respond to Google forms on computer screens at the CH and Outpatient Clinics, and face-to-face.

The groups of outpatients were interviewed face-to-face. The study was performed between September 2016 and April 2022.

### Measures

#### Demographics and Clinics Variables

The instrument consisted of the following independent sociodemographic variables: age, sex, civil status, employment status (unemployed: retired, housewife, student, or unemployed patients), and education.

#### Measuring Religiosity

The Duke University Religiosity Index (DUREL) was used to measure religious involvement. This index has five items on the three main dimensions of religiosity related to health outcomes: organizational religious activity (ORA), non-organizational (NORA), and IR refers to the internalization and full experience of religiosity as the main individual’s goal (Lucchetti et al., 2012). According to a previous study, DUREL can be used as a continuous score in each domain or as a categorical division (Stroppa & Moreira-Almeida, 2013). In this research, IR was chosen as the main religiosity dimension. Additionally, participants reported their self-declared religion at the time of the study.

#### Measurement of depression, anxiety, and stress

The Depression Anxiety Stress Scale (DASS) was developed by Lovibond and Lovibond (1995) and validated by Vignola and Tucci (2014). A self-report measure, it contains three subscales related to stress, anxiety, and depression, each containing 7 items. Responses are recorded on a 3-point Likert scale. Each subscale has a degree of severity relative to the population.

#### Sample size

The present study consisted of a secondary analysis of data collected in a larger format. In any case, the robust sample size of this study is relevant when considering the intention to investigate the importance of religiosity and its relationship with aspects of mental health among different groups. The sample size calculation considered an a priori coefficient of determination, a linear regression model with 5 predictors with significance level or type I error of *α* = 0.01, and type II error of *β* = 0.01, resulting in an a priori statistical power of 90%. Using the PASS (Power Analysis and Sample Size) application, version 13, introducing the values described above, a minimum sample size of *n* = 206 subjects for each group (outpatients and healthcare professionals) is obtained. The main outcome variable was religiosity.

#### Data Analysis

The analysis was performed using the SPSS version 23.0. Frequencies of socio-demographic variables (categories and subcategories) were performed using chi-square or Fisher’s exact tests. The Kolmogorov–Smirnov test evaluated the normality distribution of the sample. The *t* test was used to analyze continuous normally distributed variables when comparing two independent groups, and the Mann–Whitney U test was used for data not adhering to normal distribution. Bivariate correlation analysis was used to investigate the relationship between stress, anxiety, depression, sociodemographic, and religiosity variables within each group. Finally, linear regression model was performed to examine the association between IR, socio-demographic data, stress, anxiety, and depression. A significance level considered was *P* < 0.05.

#### Linear Regression Mode

Significant associations identified in bivariate correlation analyses were considered in a theoretical linear regression model to examine the relationships between IR and stress, anxiety, and depression in each population.

Stress, anxiety and depression were evaluated as dependent variables controlling for variables associated in the bivariate correlation analysis (Pearson or Spearman, *P* ≤ 0.05). Considering high collinearity statistics, in the three dimensions of religiosity (ORA, NORA and IR), IR was the main variable.

## Results

The study sample comprised 1.854 adults, 78.7% of women, mean age of 44 years old, 34% had less than eight years of formal education and 50.9% were single, separated or widowed. The majority (93.5%) reported having religious affiliation; 37.3% declared Catholic affiliation, 27% were Spiritists, 23% Evangelical/Protestant, 6.5% none, 4.4% of the Umbanda, 1.1% had other and, finally, 0.9% declared to have 2 or more religious.

In outpatients, 75% were women. Around 43% of patients were employed, and 46% were married. Almost half (47%) had less than 8 years of education. In relation to the clinical treatment department, 28.3% were in endocrinology, 16.5% in internal medicine, around 13% in the gastroenterology, among others (Table 1).

**Table 1:** Distributions of sociodemographic data of outpatients and HCP (*N* = 1.854)

Sociodemographic Data	Outpatients (*N* = 1.269)	HCP (*N* = 585)
	*M* ± SD	*M* ± SD
*Age (years)*	46.4 ± 14.9	39.1 ± 9.9
	N (%)	N (%)
Women	957 (75.4)	502 (68.8)
*Civil status*		
Single, separated or widowed	681 (53.7)	263 (45.0)
Married	587 (46.3)	322 (55.0)
*Education (years of education)*		
≤ 8	593 (46.7)	41 (7.0)
≥ 9	676 (53.3)	544 (93.0)
*Employment status*		
Employed	542 (42.7)	585 (100.0)
Unemployed	727 (57.3)	
*Clinical department*		
Internal Medicine	209 (16.5)	–
Cardiology	13 (1.0)	–
Pulmonology/thoracic	5 (0.4)	–
Neurology	55 (4.3)	–
Psychiatry	104 (8.2)	–
Endocrinology	359 (28.3)	–
Orthopedics/traumatology	121 (9.5)	–
Rheumatology	112 (8.7)	–
Cancer	104 (8.2)	–
Gastroenterology	162 (12.8)	–
Infectious & Parasitic Diseases	3 (0.2)	–
+ 1 Clinic Department	22 (1.7)	–
*Profession*		
Health technicians	–	248 (42.4)
Nurse	–	117 (20.0)
Outpatient and inpatient care	–	89 (15.2)
Rehabilitation (Physical Therapy/Speech/Occupational therapy)	–	40 (6.8)
Psychology	–	3 (0.5)
Management	–	2 (0.3)
Doctors/clinical residents	–	12 (2.1)
Nutrition	–	15 (2.6)
Students/Residents	–	6 (1.0)
Pharmacy	–	11 (1.9)
Radiology/laboratory/ occupational safety technicians	–	16 (2.7)
Social Work	–	8 (1.3)
Others	–	18 (3.2)
*Religious affiliation*		
Catholic	450 (35.5)	197 (33.6)
Evangelical/Protestant	300 (23.6)	29 (5.0)
Spiritist	304 (24.0)	226 (38.6)
Umbanda	50 (3.9)	37 (6.4)
None	72 (5.7)	59 (10.0)
Others	89 (7.0)	12 (2.1)
+ 2	4 (0.3)	25 (4.3)

*Note: The terms in italics represent subcategories of the variables analyzed. HCP* health care professionals, + *1 Clinic Department* adults undergoing 2 or more clinical departments*,* + *2* adults who declared 2 or more religious affiliations

Among HCP, 55% were women who were married, and only 7% had less than 8 years of education. The majority (62%) were nurses and health technicians. Of the total, 90% reported having religious affiliation (Table 1).

The sociodemographic characteristics in outpatients and HCP are presented in Table 1.

Of the total, 74% had stress scores, of which 56.2% were severe; 68.5% had signs of anxiety, 48.5% of which were severe; and finally, 63.2% had depressive symptoms, of these 43.7% showed severe degree.

In outpatients, around 76% showed stress, 73% had anxiety, and 68% had depression. Among HCP, approximately 72% had stress, 60% showed anxiety, and 53% had depression symptoms (see Table 2).

**Table 2:** Frequency of stress, anxiety, depression in outpatients and HCP (*N* = 1.854)

Variables	Outpatients (*N* = 1.269)	HCP (*N* = 585)
	*N* (%)	*N* (%)
*Stress*		
No	308 (24.3)	166 (28.3)
Yes	961 (75.7)	419 (71.7)
*Severe stress*		
No	537 (42.3)	267 (46.0)
Yes	732 (57.7)	316 (54.0)
*Anxiety*		
No	344 (27.1)	236 (40.3)
Yes	925 (72.9)	349 (59.7)
*Severe anxiety*		
No	617 (48.6)	335 (57.3)
Yes	652 (51.4)	250 (42.7)
*Depression*		
No	402 (31.7)	274 (46.8)
Yes	867 (68.3)	311 (53.2)
*Severe depression*		
No	675 (53.2)	364 (62.3)
Yes	594 (46.8)	221 (37.7)

*Note: The terms in italics represent subcategories of the variables analyzed. HCP* health care professionals

In bivariate correlation analysis of outpatients, variables associated with stress were IR (*r* =  − 0.14, *p* = 0.000), age (*r* =  − 0.20, *p* = 0.000), sex (*r* = 0.15, *p* = 0.000), and education (*r* = 0.10, *p* = 0.006); variables associated with anxiety were IR (*r* =  − 0.09, *p* = 0.001), age (*r* =  − 0.16, *p* = 0.000), sex (*r* = 0.19, *p* = 0.000), and employment status (*r* = 0.07, *p* = 0.030); and variables associated with depression were IR (*r* =  − 0.16, *p* = 0.000), age (*r* =  − 0.12, *p* = 0.000), sex (*r* = 0.19, *p* = 0.000), and education  (*r* = 0.13, *p* = 0.000).

The analyses of HCP showed that ORA (*r* =  − 0.20, *p* = 0.047), NORA (*r* =  − 0.24, *p* = 0.019), IR (*r* =  − 0.23, *p* = 0.027), age (*r* = − 0.15, *p* = 0.001), and profession (*r* =  − 0.14, *p* = 0.001) variables are associated with stress; sex (*r* = 0.11, *p* = 0.004), and profession (*r* =  − 0.14, *p* = 0.000) were associated with anxiety; and variables associated with depression were IR (*r* = 0.16, *p* = 0.045), sex (*r* = 0.10, *p* = 0.014), and profession (*r* =  − 0.13, *p* = 0.001).

Linear regression analysis showed a statistical association between higher IR with less stress, anxiety, and depression, controlling for age, sex, and education variables; lower age has higher indicative scores for stress, anxiety, and depression; lower education has greater indicative scores for stress and depression; and the category unemployed is associated with higher anxiety scores in outpatients (Table 3).

**Table 3:** Multivariade linear regression model stress, anxiety, and depression scales among outpatients and HCP

	Unstandardized Coeficients	Standardized Coeficients	*t*	*Sig*	Collinearity Statistics	
	*B*	*Beta*			Tolerance	*VIF*
Outpatients						
*Stress*						
Intrinsic religiosity	− 0.40	− 0.17	− 3.76	.000**	0.91	1.09
Age	− 0.06	− 0.17	− 3.45	.001**	0.71	1.39
Sex	0.35	0.02	0.50	.613	0.98	1.01
Education	− 0.66	− 0.13	− 2.87	.004**	0.86	1.15
Civil status	− 0.06	− 0.00	− .18	.853	0.77	1.28
Employment status	0.40	0.02	0.59	.555	0.91	1.09
*Anxiety*						
Intrinsic religiosity	− 0.31	− 0.14	− 3.05	.002**	0.91	1.09
Age	− 0.05	− 0.13	− 2.57	.010*	0.71	1.39
Sex	1.07	0.07	1.56	.119	0.98	1.01
Education	− 0.37	− 0.07	− 1.66	.097	0.86	1.15
Civil status	0.10	0.01	.33	.735	0.77	1.28
Employment status	1.41	0.09	2.14	.033*	0.91	1.09
*Depression*						
Intrinsic religiosity	− 0.49	− 0.21	− 4.82	.000**	0.91	1.09
Age	− 0.06	− 0.16	− 3.18	.002**	0.71	1.39
Sex	0.98	− 0.06	1.45	.148	0.98	1.01
Education	− 0.53	− 0.11	− 2.38	.017*	0.86	1.15
Civil status	0.10	0.01	0.31	.752	0.77	1.28
Employment status	1.01	0.07	1.54	.123	0.91	1.09
HCP						
*Stress*						
Intrinsic religiosity	− 1.09	− 0.22	− 2.11	.037*	0.99	1.01
Age	− 0.13	− 0.13	− 1.29	.200	0.98	1.01
Profession	− 0.33	− 0.03	− 0.29	.767	0.97	1.02
*Anxiety*						
Sex	2.40	0.11	2.72	.007**	1.00	1.00
Profession	− 0.34	− 0.14	− 3.55	.000**	1.00	1.00
*Depression*						
Intrinsic religiosity	− 0.59	-0.15	− 1.99	.048*	0.99	1.00
Sex	6.60	.024	3.08	.002**	0.98	1.01
Profession	1.29	.010	1.27	.204	0.98	1.01

Note: The terms in italics represent subcategories of the variables analyzed. The values ​​in italics indicate statistically significant results.                                                                                                                 Outpatients: Dependent coefficient variables: stress scale (*N* = 1.269, *R square* 0.08, adjusted *R square* 0.07; *p* = .000); anxiety scale (*N* = 1.269, *R square* .06, adjusted *R square* 0.04, *p* = .002); depression scale (*N* = 1.269, *R square* 0.10, adjusted *R square* 0.08, *p* = .000);

*HCP* health care professionals: Dependent coefficient variables: stress scale (*N* = 585, *R square* 0.06, adjusted *R square* 0.03; *p* = .098); anxiety scale (*N* = 585, *R square* 0.03, adjusted *R square* 0.03, *p* = .000); depression scale (*N* = 585, *R square* 0.10, adjusted *R square* 0.08, *p* = .002);

^**^p < 0.01; *p < 0.05

In HCP, a linear regression model identified a statistical association between higher IR with less stress, controlling for age and profession. The model adjusted R Square showed an association between lower IR and women with higher depression, controlling for profession. Women have higher indicative scores for anxiety and depression than men, and nurses and health technicians have higher indicative scores for anxiety (Table 3).

## Discussion

The present study demonstrated that higher IR is associated with less stress, anxiety, and depression in outpatients, and it showed associations with lower stress and depression in a sample of HCP. Our findings are in accordance with studies reporting a protective effect of R/S on stress, depression, chronic diseases and adversities (Smit et al., 2003; Paglione et al., 2019; Mosqueiro et al., 2021; Carneiro et al., 2018; Rezaeipandari et al., 2023) and religiosity can alleviate anxiety (de Diego-Cordero et al., 2022; Fenelon & Danielsen, 2016; Lucchetti et al., 2021; VanderWeele et al., 2017).

Participants who reported higher IR had lower stress and depression symptoms. Depression has been identified as one of the leading causes of the disease burden worldwide. Depressive disorders are highly prevalent, disabling, and costly. They are linked with considerably medical comorbidity that decreases quality of life and increases mortality. Identification of the potential factors that decrease the risk of depression could be important to provide prevention strategies (Cuijpers et al., 2014; Demyttenaere et al., 2004; Steel et al., 2014). Religiosity is a desirable coping strategy for many people when facing negative life events such as infirmities and stressors. Recent reviews show that effects of R/S on mental health are likely bidirectional, and the manner in which religious beliefs are used to cope with distress (negative and positive) may affect mental health outcomes (Balboni et al., 2022; Lucchetti et al., 2021).

Recent study found that religious behaviours and the possession of sincere beliefs based on religion can be considered mechanisms used to better cope with their illnesses (Rezaeipandari et al., 2023).

Corroborating our findings, IR was consistently associated with positive mental health outcomes in depressed inpatients (Mosqueiro et al., 2021). A study using the latest mental health cycle of the Canadian Community Health Survey (*N* = 20.868) examined the importance of R/S in one’s life associated with mental health and identified that highly religious individuals classify their mental health as excellent (Dilmaghani, 2018).

Furthermore, younger outpatients have higher indicative scores for stress, anxiety, and depression; fewer years of study have greater scores for stress and depression; and being employed is associated with lower indicative scores for anxiety. In HCP, women have greater indicative scores for anxiety and depression than men, and nurses/health technicians have higher indicative scores for anxiety. Epidemiological sex differences in anxiety disorders and major depression are well characterized (Bangasser & Cuarenta, 2021). Meta-analysis corroborates our findings and verifies that burnout syndrome had a significant relationship with depression in nurses (Ramírez-Elvira et al., 2021). Future studies can explore the mechanism of IR influence on mental and physical health.

### Study limitations

The present study has several limitations. First, study populations were subjected to different data collection methods. Live responses to an interviewer, even when duly trained not to interfere in the responses, may be considered a source of bias. Another limitation of the present study, because of its cross-sectional methodology, is the impossibility of evaluating the impact of religiosity on mental health changes over time.

## Conclusions

Regardless of the group they were in, IR was found to be associated with less stress and depression symptoms. In addition, IR is associated with less anxiety symptoms in outpatients. These findings support the relevance of R/S for improving mental health in outpatients and HCP. The HCP should be aware of the beliefs of their patients, address R/S in clinical practice, and managers can provide R/S strategies to HCP in hospitals and outpatient clinics.
